# Development and Validation of an LC–MS/MS Method for Quantifying Gabapentin in Plasma: Application to a Pharmacokinetic Study in Cats

**DOI:** 10.3390/ani15070950

**Published:** 2025-03-26

**Authors:** Feifei Zhao, Changcheng Lin, Yunying Wu, Xinyue Luo, Ning Han, Wenguang Xiong, Zhenling Zeng

**Affiliations:** 1 Guangdong Provincial Key Laboratory of Veterinary Pharmaceutics Development and Safety Evaluation, College of Veterinary Medicine, South China Agricultural University, Guangzhou 510642, China; ffzhao@stu.scau.edu.cn (F.Z.);; 2 National Risk Assessment Laboratory for Antimicrobial Resistance of Animal Original Bacteria, Guangzhou 510642, China

**Keywords:** gabapentin, LC-MS/MS, cat plasma, pharmacokinetics

## Abstract

Gabapentin (GBP), a γ-aminobutyric acid analogue used for neuropathic pain and epilepsy, can reduce cat stress and improve veterinary exam compliance. A sensitive LC-MS/MS assay was established and rigorously validated for quantifying GBP in feline plasma. The method employed acetonitrile-mediated protein precipitation to efficiently extract GBP and its internal standard, pregabalin (PGB). Chromatographic separation was achieved within 7 min using a C18 column (2.1 × 50 mm, 1.7 μm) with a gradient mobile phase comprising 0.1% (*v*/*v*) formic acid in water and acetonitrile. The pharmacokinetics of 25  mg/kg GBP was studied by single-dose oral and intravenous administration. The results demonstrated that the method exhibited satisfactory precision, accuracy and linearity. The pharmacokinetic results showed that the T_max_, C_max_, T_1/2_ and AUC_0–t_ of GBP in cats after oral administration of 25  mg/kg were (1.83 ± 0.75) h, (13.94 ± 3.75) μg/mL, (5.60 ± 1.79) h and (115.54 ± 27.56) (μg/mL) h, respectively. The results of the study indicated that after intravenous administration of 25 mg/kg GBP, the C_0_, T_1/2_ and AUC_0–t_ were (58.82 ± 15.34) μg/mL, (3.87 ± 0.64) h and (160.44 ± 32.65) (μg/mL) h, respectively. The oral bioavailability of GBP in cats was (78.71 ± 18.55)%. In this study, a selective and sensitive LC-MS/MS method for the quantification of GBP in cat plasma was developed and validated. This method was successfully employed to assess the pharmacokinetics following the oral and intravenous administration of GBP in cats.

## Simple Summary

Gabapentin is an analogue of the neurotransmitter γ-aminobutyric acid, which is commonly used to treat neuropathic pain and epilepsy. However, studies have shown that early use of gabapentin can significantly reduce cats’ stress and increase their compliance with veterinary examinations. There is currently little research on the pharmacokinetics of gabapentin tablets for oral administration in cats. Consequently, monitoring gabapentin plasma levels provides critical insights into therapeutic adherence and facilitates early identification of toxicity thresholds. In this study, a novel LC-MS/MS assay was established and comprehensively validated to achieve high-throughput quantification of gabapentin in feline plasma resolution within 7 min. Additionally, this study provides further clinical evaluation for the management of sedation and anti-stress in cats.

## 1. Introduction

Cats are the most popular pets in Canada and Europe, and are ranked the second most popular pets in the world [1]. Although cats are popular, they face barriers in accessing veterinary care, especially compared to dogs. These obstacles include stress during transportation and treatment, as well as the owner’s lack of understanding of preventive care and difficulty in identifying symptoms of disease [2,3]. Despite the recommendation of numerous non-drug strategies to reduce fear and stress in cats during treatment, some cats still exhibit fear and aggressive behavior [4]. In such cases, drug intervention prior to handling the cats may be considered in order to reduce cats’ stress and facilitate their adaptation to the external environment and staff [5,6]. The evidence base for establishing standardized oral sedation protocols in felines is currently insufficient, necessitating a pharmacokinetic model to bridge this gap.

GBP has recently gained popularity as a pharmaceutical agent for the treatment of anxiety and pain. Clinical studies have demonstrated its efficacy in alleviating anxiety and stress in felines. An investigation revealed that oral administration of 100 mg GBP capsules to cats 90 min prior to their transportation to a veterinary facility and subsequent examination significantly reduced stress-related behaviors during transportation and examination, as well as instances of feline aggression during the veterinary examination. Additionally, the administration of GBP was observed to alleviate acute stress in cats induced by veterinary intervention [7,8,9].

Gabapentin, a γ-aminobutyric acid (GABA) structural derivative, selectively binds to the α2δ-1 subunit of voltage-gated calcium channels—the canonical receptor mediating thrombospondin-dependent synaptogenesis [10,11]. Its pharmacological activity primarily involves antagonism of central voltage-gated Ca^2+^/Na^+^ channels via presynaptic membrane potential modulation, concomitantly with suppressed monoamine exocytosis, attenuated excitatory synaptogenesis and potentiation of GABAergic/serotonergic neurotransmission [12]. It is commonly used in human medicine for the treatment of chronic pain conditions that are maladaptive in nature, such as diabetic neuropathy [13]. Currently, the drug has been approved by the United States Food and Drug Administration (USFDA) for the treatment of postherpetic neuralgia and as an adjunctive therapy for partial-onset seizures in humans.

GBP has been utilized to control seizures, anxiety in companion animals and neuropathic pain in humans, rats and cattle [14,15,16]. Although the pharmacokinetics of GBP in cats following oral administration have been reported, its bioavailability may show great inter- and intra-subject variability, because of its particular active absorption by the gut and excretion by the kidneys [17]. For this reason, the determination of blood concentrations of GBP may be useful in assessing compliance and evaluating the risk of toxicity. Additionally, this study provides further clinical evaluation for the management of sedation and anti-stress in cats.

At present, the determination methods for the plasma concentration of GBP and PGB include HPLC [18,19,20], LC-FL [21], LC-MS [11,13], etc. However, these two drugs do not have ultraviolet absorption, and require pre-column derivatization, which leads to complex pretreatment of plasma samples and long detection times. No single method has been reported that can simultaneously determine GBP and structurally similar compounds, like PGB, without derivatization. Therefore, the LC-MS/MS-based quantitative analytical method established for determining GBP in feline plasma fulfills requirements for rapid, accurate and high-throughput detection, while elucidating the correlation between the pharmacokinetic profile of this drug in cats and its clinical applicability in mitigating stress-related responses during veterinary interventions. Furthermore, the notably high oral bioavailability of GBP in cats may be attributed to species-specific gastrointestinal absorption characteristics and hepatic metabolic enzyme activity, which provides a theoretical foundation for developing optimized sustained-release formulations tailored to feline pharmacokinetic parameters.

## 2. Materials and Methods

### 2.1. Animals

The cats included in this study were healthy adults weighing 3~5 kg and of appropriate age, confirmed by veterinary examination during a 7-day pre-trial quarantine. Excluded cats were those with intercurrent diseases or those not meeting the weight/age criteria. Therefore, twelve healthy adult shorthair cats, half male and half female, weighing an average of 3.91 kg (range: 3.47~4.59 kg), were used in the study. Cats were allowed ad libitum access to food and water during the study. The study was reviewed and approved by the Institutional Animal Care and Use Committee of South China Agricultural University (approval number: 2023A022).

### 2.2. Chemical and Standards

Gabapentin (99.8% purity) and its respective internal standard pregabalin (99.4% purity) were obtained from the National Institutes for Food and Drug Control. GBP tablets (100 mg specification) and injections (50 mg/mL specification) were provided by Shanghai Hanvet Biomedical Technology Co., Ltd. (Shanghai, China). All solvents and reagents were procured from certified suppliers: mass spectrometry-grade acetonitrile and methanol were sourced from Fisher Scientific (Waltham, MA, USA), and spectrometry-grade formic acid was sourced from Aladdin Biochemical Technology Co., Ltd. (Shanghai, China).

### 2.3. Calibration Standards and Quality Control

Stock solutions of GBP and PGB were made by dissolution in methanol. Working solutions were prepared by diluting the stock solution in acetonitrile. To prepare calibration and quality control (QC) samples, working solutions were added to blank plasma. GBP calibration samples covered a range from 50 ng/mL to 5000 ng/mL. Additionally, QC samples were prepared at the limit of quantification (LLOQ: 50 ng/mL) and at low- (LQC: 100 ng/mL), medium- (MQC: 500 ng/mL) and high-quality concentrations (HQC: 4000 ng/mL), by adding a blank of cat plasma.

### 2.4. Sample Preparation

A 200 μL volume of cat plasma (Eppendorf, Hamburg, Germany) was placed in a 2 mL centrifuge tube, followed by the addition of 10 μL of PGB working solution (10.5 μg/mL), together with 390 μL acetonitrile. After vortexing, the samples were centrifuged at 10,000 rpm for 10 min, at 4 °C (Thermo, Waltham, MA, USA). Subsequently, 300 μL of supernatant was mixed with 700 μL ultrapure water, filtered through a 0.22 μm membrane for concentration analysis.

### 2.5. LC-MS/MS Instrumentation and Setting

The analysis was performed using a Nexera XR ultra-high performance liquid chromatography system (Shimadzu Corporation, Kyoto, Japan), interfaced with an LCMS-8050 triple-quadrupole mass spectrometer (Shimadzu, Kyoto, Japan) equipped with an electrospray ionization (ESI) source. Analytical detection was conducted in positive ionization mode, with the following optimized parameters: ion source temperature—300 °C, desolvation line temperature—526 °C, and nebulizing gas flow rate—3.0 L/min. Instrument control and data processing were managed through LabSolutions Insight software (version 5.99, Shimadzu).

Chromatographic separation was performed on a Phenomenex Luna C18 column (Phenomenex, Torrance, CA, USA) (150 mm × 2 mm, 5.0 μm), using a gradient program, at a flow rate of 0.3 mL/min and an injection volume of 3 µL, with the column temperature at 40 °C. Briefly, chromatographic separation was achieved using acidified (0.1% formic acid) water/acetonitrile mixtures in gradient elution mode. The gradient elution program was optimized as follows: 10% B at 0~1.0 min, 10~80% B at 1.0~2.0 min, 80% B at 2.0~4.0 min, 80~10% B at 4.0~4.5 min, and 10% B at 4.5~7.0 min.

### 2.6. Method Validation

Verification was performed following the European Medicines Agency (EMA) and the US Food and Drug Administration (FDA) bioanalytical method validation guidelines. The parameters verified were linearity, matrix effect, accuracy and precision, stability and dilution integrity.

#### 2.6.1. Selectivity and Specificity

Plasma samples from healthy trial cats were collected, including blank samples, post-administration samples and blank samples spiked with GBP. This process aimed to compare the interference of impurities in GBP across different blank plasma sources.

#### 2.6.2. Linearity

Standard curves of the samples were prepared in accordance with the protocols described in Section 2.3. A linear calibration curve was constructed using a 1/x^2^ weighting factor. Six calibration points were used, and the curve was created by calculating the ratio of the analyte peak area to the IS peak area (r^2^ > 0.99).

#### 2.6.3. Accuracy and Precision

Accuracy and precision were measured in three individual analytical runs on three different days. A total of six replicates were evaluated for each QC level, namely LLOQ, LQC, MQC and HQC. The intra- and inter-assay accuracy of QC samples of one level was required to be 85~115% (80~120% for the LLOQ). To assess precision, the coefficient of variation (CV = standard deviation/mean × 100%) was employed. The intra- and inter-assay CV needed to be <15% (<20% for the LLOQ).

#### 2.6.4. Matrix Effect

The matrix effect was determined as the ratio of the peak area of the analyte with the matrix to the peak area of the analyte without the matrix. The matrix effect factor (MF) and the matrix effect factor normalized to the internal standard (MFi) were calculated using the following formulas. It was determined that the coefficient of variation for the MFi calculated from six batches of matrix should be less than 15%.

Matrix effect factor (MF) = $\frac{\text{Analyte peak area in plasma}}{\text{Analyte peak area in neat solution}}$.

Matrix effect factor normalized to the internal standard (MFi) = $\frac{\text{Absolute matrix factor of the analyte}}{\text{Absolute matrix factor of the internal standard}}$.

#### 2.6.5. Stability

The stability of the GBP (LQC and HQC) was measured under different conditions: after short-term storage at room temperature for 24 h, after long-term storage at −20 °C for 30 d, after 3 freeze–thaw cycles (from −20 °C to room temperature) and after being placed in an autosampler at 4 °C for 30 h. Furthermore, analytes were considered stable when the measured concentrations were within ±15% of the baseline HQC and ±20% of the baseline LQC. All QC levels were measured in triplicate.

#### 2.6.6. Carryover

The carryover was estimated by injecting blank samples after HQCs were injected. More importantly, it was determined that the peak areas in the blank samples after injection of the HQCs should not exceed 20% of the LQC, and should not exceed 5% of the IS.

#### 2.6.7. Dilution Integrity

The integrity of the dilution was demonstrated by adding the analyte to the matrix to a concentration above the ULOQ, and diluting the sample with blank plasma (1:5 or 1:10). It was established that the accuracy and precision should be within ±15%. Furthermore, it was confirmed that the reliability of dilution should cover the test samples.

### 2.7. Pharmacokinetics

The validated method was used for the pharmacokinetic study of GBP tablets in healthy cats. Twelve cats were randomly divided into two groups, and we confirmed that the groups were well matched through randomization. The cats were fasted for 12 h before administration and were free to drink water. Six cats were administered a single oral dose of 25  mg/kg GBP tablets, while the remaining cats received a single intravenous dose of 25  mg/kg GBP injection. Blank blood was collected from the antecubital vein into an anticoagulant tube containing heparin sodium before administration. Subsequent blood samples were collected at 0.25, 0.75, 1, 2, 3, 4, 5, 6, 9, 12, 16, 24, 36, 48 and 60 h after oral administration of GBP. Additionally, intravenous plasma samples were collected at 0.08, 0.17, 0.25, 0.50, 1, 2, 4, 6, 8, 12, 16, 24, 36, 48 and 60 h. All blood samples were centrifuged (Thermo, Dreieich, Germany) at 3500 rpm, at 4 °C, for 15 min to separate the plasma, and stored in a −20 °C refrigerator until analysis. The plasma concentration–time data for each cat were plotted to generate individual and average pharmacokinetic curves. Subsequently, Phoenix WinNonlin^®^ 8.2 software (Certara, Princeton, NJ, USA) was employed to process and statistically analyze the measured pharmacokinetic data via non-compartmental model, to comprehensively understand the drug’s absorption, distribution, and elimination in cats.

## 3. Results

### 3.1. LC-MS/MS Method Development

**Figure 1:**
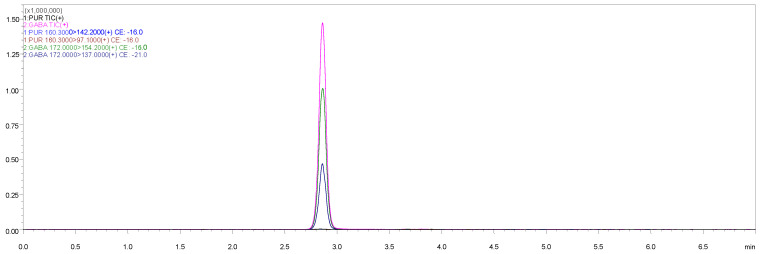
Chromatogram of 50 ng/mL GBP standard working solution.

Two transitions per analyte were selected for quantification and confirmation. Table 1 presents the MRM transitions and MS settings applied for the analytes in this method. The representative chromatogram is shown in Figure 1.

### 3.2. Method Validation

#### 3.2.1. Selectivity and Specificity

**Figure 2:**
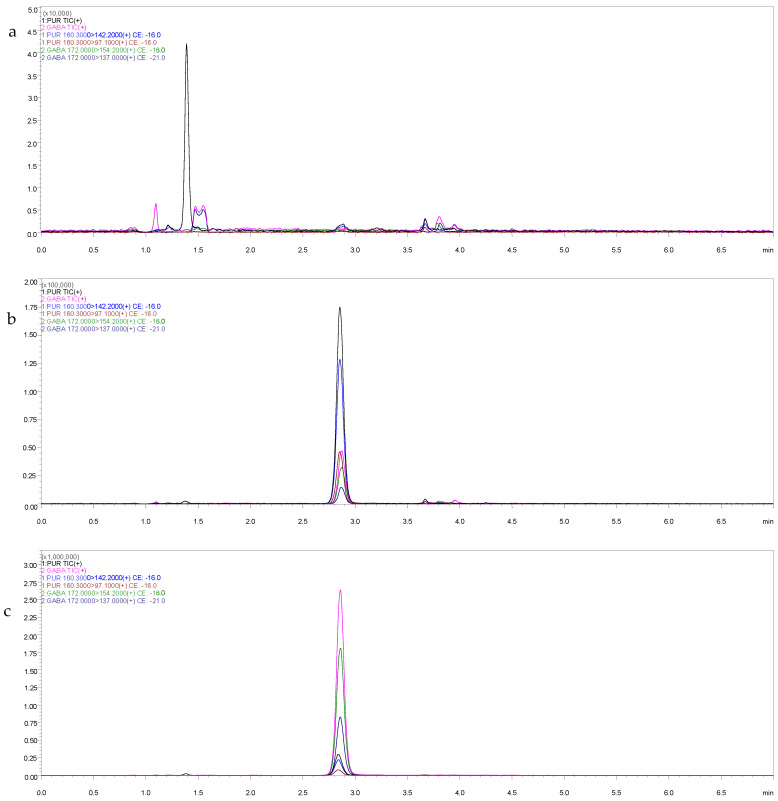
Chromatogram of (**a**) blank cat plasma sample, (**b**) GBP added to plasma (added concentration 50 ng/mL) and (**c**) plasma sample of cat 1 h after taking GBP tablet (100 mg) orally.

Samples of blank plasma, blank plasma with GBP and PK plasma were determined. The results show that the samples were not affected by impurities in the plasma. The drug and impurity peaks are clearly separated, indicating that the method was selective and specific. As shown in Figure 2, the GBP and internal standard (PGB) both elute consistently at around 2.9 min.

#### 3.2.2. Linearity

The developed method showed a linear relationship in the range of 50~5000 ng/mL of GBP. A weighting factor of 1/x^2^ was applied. All coefficients of determination (r^2^) for all validated batches were greater than 0.99. The limit of deviation from the nominal value was ≤15% (≤20% for the LLOQ). Moreover, the LOD and LOQ, based on the signal-to-noise ratio of three (S/N = 3) and ten (S/N = 10), were within 20 ng/mL and 50 ng/mL ranges, respectively.

#### 3.2.3. Accuracy and Precision

For all QCs, the intra-day and inter-day precision and accuracy met the defined criteria (LLOQ was 50 ng/mL, with acceptable accuracy (93%) and precision; QCs within 15%) in Table 2.

#### 3.2.4. Matrix Effect

The IMF (Internal Matrix Factor) of six different plasma sources determined that the LQC and HQC levels were 104.68% and 101.44% for GBP, and the CVs of LQC and HQC were 3.43% and 3.03%, respectively. The matrix effect had a negligible impact in this method.

#### 3.2.5. Stability

As shown in Table 3, the results of the three freeze–thaw cycles and short-term, long-term and processed sample stability studies revealed that all QCs were found to be stable.

#### 3.2.6. Carryover

No carryover was observed for GBP and PGB. The carryover percentages were all below 2.76%. Also, no LOQ peak area was more than 20% of the reference peak area and 5% of the reference IS peak area.

#### 3.2.7. Dilution Integrity

The CVs of 5-fold and 10-fold diluted QCs of GBP were 3.65% and 3.82%, respectively. The results indicate that the dilution procedures did not affect the accuracy and precision of the measured concentration of GBP.

### 3.3. Pharmacokinetics

**Figure 3:**
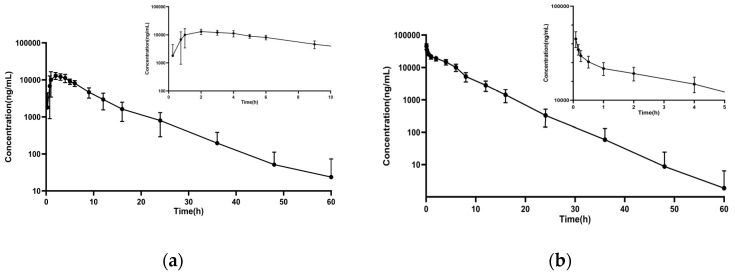
Plasma concentrations of GBP over 60 h (**a**) after oral administration of 25 mg/kg in healthy cats, and (**b**) after intravenous administration of 25 mg/kg in healthy cats. Data expressed as mean ± SD (n = 6).

The validated LC-MS/MS method was effectively used to analyze clinical plasma samples from 12 cats after single oral and intravenous doses of 25 mg/kg GBP. The concentration–time curves of GBP are shown in Figure 3, and the corresponding pharmacokinetic parameters are listed in Table 4.

## 4. Discussion

The majority of existing bioanalytical methods for the analysis of GBP in plasma require the use of time-consuming sample preparation techniques, such as solid-phase extraction [11,22] or evaporation [13,23,24] steps. The objective of this study was to develop a bioanalytical method for rapid sample analysis and successfully apply this to the pharmacokinetic study of cats.

Initially, the analyte and IS were injected into the mass spectrometer to optimize the ionization parameters and detect the fragmentation pattern. All analytes were detected in positive ion mode [25]. The optimization of chromatographic conditions involved choosing a proper mobile phase and chromatographic column. According to reported research [26], adding certain proportion of formic acid into the mobile phase could improve the ionization efficiency of analytes and effectively improve the peak shape. Consequently, 0.1% formic acid/water-acetonitrile was selected as the mobile phase. Furthermore, in order to separate the tested compounds and obtain sharp peak shapes, the gradient elution mode was optimized. Phenomenex Luna C18 (150 mm × 2 mm, 5 μm) demonstrated excellent separation and symmetry; further evaluation was not deemed necessary.

The method used in this study was optimized on the basis of previous methods. The efficacy of direct precipitation of proteins in samples using acetonitrile and methanol was evaluated [27]. The results showed that acetonitrile was better than methanol for protein precipitation, and had a better peak shape.

The AUC_0–t_ values of oral and intravenous GBP were 115.54 ± 27.56 (μg/mL) h and 160.44 ± 32.65 (μg/mL) h, respectively. T_max_ reflects the rate of absorption of a drug. The absorption observed in this study (T_max_ 1.83  ±  0.75  h) was faster than that noted in humans (6.9  ±  2.1  h) and goats (8.47 ± 1.9 h), but similar to what has been observed in cats (1.67 ± 0.37 h), horses (1.41  ±  0.1  h) and dogs (1.51 ± 0.5 h) [11,22,27,28,29]. The terminal half-life (t_1/2_) for IV GBP in the current study (3.87  ±  0.64 h) was slightly longer than that reported in dogs (2.9 h), but shorter than that reported in horses (8.53 h) [30,31].

The bioavailability of a drug is defined as the proportion of the active ingredient that reaches systemic circulation. An absolute bioavailability of greater than 50% is generally considered to indicate high oral bioavailability [32]. The bioavailability was calculated between different individuals, which may have introduced variability. We compared our pharmacokinetic findings with prior feline studies. Our results showed that the Tmax and bioavailability were 1.83 h and 78.71%, which are consistent with previous feline studies [11,13]. In addition, the bioavailability of GBP in humans (74.1%) and dogs (80%) is comparable to what we reported, but significantly higher than that in horses (16.2%) [29,30,31]. Furthermore, the half-life we observed was 5.6 h, which is different to that in previous reports. These differences may be due to factors such as variations in study design, cat breeds, ages or the specific formulations of GBP used.

Also, we chose a parallel-group design instead of a crossover design for several reasons. First, the parallel-group design was particularly suitable for our study objectives, as it allows for independent comparison between different groups without the potential carryover effects that can occur in a crossover design. Second, considering the welfare of the cats, whose health could potentially have been affected due to the volume of blood collected, the parallel-group design minimized this concern by distributing the blood collection across different groups, thereby reducing the burden on individual animals. We acknowledge that the parallel-group design may have certain limitations compared to a crossover design, such as requiring a larger sample size and potentially being more resource-intensive. However, we believe that the benefits of this design outweigh these limitations in our specific study context. Regarding the matching of groups, we employed a rigorous randomization process to ensure that the groups were well matched at baseline.

The preclinical efficacy of GBP in chronic pain management has been extensively validated in various animal models. In a rat model of thermal hyperalgesia induced by burn injury, intraperitoneal administration of GBP significantly alleviated mechanical allodynia, demonstrating dose-dependent analgesic effects [33]. Similarly, in formalin-evoked inflammatory pain models, GBP synergized with ibuprofen to enhance pain relief, suggesting its potential for combination therapy [34]. Notably, GBP outperformed morphine and amitriptyline in reducing pain-related behaviors when administered intrathecally in neuropathic pain models, highlighting its central nervous system efficacy [35]. Studies using carrageenan-induced peripheral sensitization have further confirmed gabapentin’s ability to suppress nociceptive processing, particularly through modulation of voltage-gated calcium channels and NMDA receptor antagonism [36]. These findings across diverse models—including thermal, chemical and neuropathic pain paradigms—consistently support GBP’s clinical translatability for chronic pain conditions [37,38].

In addition, our results indicate that GBP reached its maximum concentration (Cmax) at 1.83 h; this rapid absorption aligns with clinical protocols that administer GBP 1~2 h pre-appointment to reduce feline stress. Likewise, the lack of adverse effects at 25 mg/kg could be mentioned as reassuring for using such doses for stress reduction. These findings provide evidence-based support for timing GBP administration to achieve effective anxiety and stress reduction in veterinary settings.

## 5. Conclusions

This study successfully developed and verified an LC-MS/MS method suitable for the quantitative analysis of gabapentin in cat plasma. The plasma sample pretreatment is simple, the detection speed is fast, the method is stable and samples can be efficiently analyzed on a large scale. The method was applied to the pharmacokinetic study of gabapentin in cats, and the results demonstrated that the absorption and elimination were slower than previously reported results in cats. Importantly, no adverse reactions were found during the trial. This provides a scientific basis for rational drug use in veterinary clinics.

## Figures and Tables

**Table 1:** Mass spectrometric conditions for each analyte.

Analyte	Precursor Ion (*m*/*z*)	Product Ion (*m*/*z*)	Collision Energy (V)
Gabapentin	172.0	154.2 ^1^/137.0	16/21
Pregabalin	160.3	142.2 ^1^/97.1	16/16

^1^ Quantitative ion used for quantification.

**Table 2:** Accuracy and precision of determination of gabapentin in cat plasma by LC-MS/MS (n = 6).

Analyte	Nominal Concentration (ng/mL)	Intra-Day (n = 6)			Inter-Day (n = 18)		
		Measured Concentration (mean ± SD, ng/mL)	Precision (CV%)	Accuracy (%)	Measured Concentration (mean ± SD, ng/mL)	Precision (CV%)	Accuracy (%)
Gabapentin	50	46.57 ± 1.76	3.45	93.15	50.67 ± 5.78	3.13	101.34
	100	94.85 ± 9.35	9.00	94.85	100.62 ± 9.61	5.99	100.62
	500	515.77 ± 30.84	5.46	103.15	521.11 ± 31.50	5.76	104.22
	4000	3660.63 ± 116.84	2.91	91.52	3620.75 ± 106.73	1.96	90.52

**Table 3:** Stability tests for gabapentin in cat plasma (n  =  6).

Analyte	Nominal Concentration (ng/mL)	Short-Term (24 h, 25 °C)		Long-Term (−20 °C, 30 d)		Freeze–Thaw Stability 3 cycles)		Autosampler (30 h, 4 °C)	
		Measured Concentration (ng/mL)	Accuracy (%)	Measured Concentration (ng/mL)	Accuracy (%)	Measured Concentration (ng/mL)	Accuracy (%)	Measured Concentration (ng/mL)	Accuracy (%)
Gabapentin	100	110.16	2.05	109.40	6.40	112.61	2.08	114.06	2.57
	4000	4405.26	4.95	4133.93	5.87	4466.99	2.82	4309.72	4.19

**Table 4:** Pharmacokinetic parameters for GBP after IV administration (25 mg/kg) or oral administration (25 mg/kg) in 12 cats.

Parameters	Unit	Oral	IV
T_1/2_	h	5.60 ± 1.79	3.87 ± 0.64
T_max_	h	1.83 ± 0.75	*
C_max_	μg/mL	13.94 ± 3.75	44.88 ± 7.82
CL/F	L/h	0.89 ± 0.17	*
V_d_/F	L	7.03 ± 2.21	*
AUC_0–t_	(μg/mL) h	115.54 ± 27.56	160.44 ± 32.65
AUC_0–∞_	(μg/mL) h	116.48 ± 27.46	161.70 ± 32.95
C_0_	μg/mL	*	58.82 ± 15.34
CL	L/h	*	0.65 ± 0.15
V_d_	L	*	3.50 ± 0.37
V_ss_	L	*	3.26 ± 0.39
F	%	78.71 ± 18.55	*

*: Not applicable for this route.

## Data Availability

The corresponding authors will make the data supporting this study available upon reasonable request.
